# Structuring osteosarcoma knowledge: an osteosarcoma-gene association database based on literature mining and manual annotation

**DOI:** 10.1093/database/bau042

**Published:** 2014-05-27

**Authors:** Kathrin Poos, Jan Smida, Michaela Nathrath, Doris Maugg, Daniel Baumhoer, Anna Neumann, Eberhard Korsching

**Affiliations:** ^1^Institute of Bioinformatics, University of Münster, Münster, Germany, ^2^Clinical Cooperation Group Osteosarcoma, Helmholtz Zentrum München, German Research Center for Environmental Health, 85764 Neuherberg, Germany, ^3^Children's Cancer Research Center and Department of Pediatrics, Klinikum rechts der Isar, Technische Universität München, 81664 Munich, Germany and ^4^Bone Tumor Reference Center at the Institute of Pathology, University Hospital Basel, Basel, Switzerland

## Abstract

Osteosarcoma (OS) is the most common primary bone cancer exhibiting high genomic instability. This genomic instability affects multiple genes and microRNAs to a varying extent depending on patient and tumor subtype. Massive research is ongoing to identify genes including their gene products and microRNAs that correlate with disease progression and might be used as biomarkers for OS. However, the genomic complexity hampers the identification of reliable biomarkers. Up to now, clinico-pathological factors are the key determinants to guide prognosis and therapeutic treatments. Each day, new studies about OS are published and complicate the acquisition of information to support biomarker discovery and therapeutic improvements. Thus, it is necessary to provide a structured and annotated view on the current OS knowledge that is quick and easily accessible to researchers of the field. Therefore, we developed a publicly available database and Web interface that serves as resource for OS-associated genes and microRNAs. Genes and microRNAs were collected using an automated dictionary-based gene recognition procedure followed by manual review and annotation by experts of the field. In total, 911 genes and 81 microRNAs related to 1331 PubMed abstracts were collected (last update: 29 October 2013). Users can evaluate genes and microRNAs according to their potential prognostic and therapeutic impact, the experimental procedures, the sample types, the biological contexts and microRNA target gene interactions. Additionally, a pathway enrichment analysis of the collected genes highlights different aspects of OS progression. OS requires pathways commonly deregulated in cancer but also features OS-specific alterations like deregulated osteoclast differentiation. To our knowledge, this is the first effort of an OS database containing manual reviewed and annotated up-to-date OS knowledge. It might be a useful resource especially for the bone tumor research community, as specific information about genes or microRNAs is quick and easily accessible. Hence, this platform can support the ongoing OS research and biomarker discovery.

**Database URL**: http://osteosarcoma-db.uni-muenster.de

## Introduction

Osteosarcoma (OS) the most common primary malignant tumor of bone frequently affects children and young adolescents (1). It is a complex disease with manifold numerical and structural genomic alterations affecting multiple genes to a varying extent (2). Patients without clinical signs of systematic spread show 5-year survival rates of 60–80% (3), whereas patients with metastasis at diagnosis exhibit 5-year survival rates of 20–30%. Since 1980, the prognosis of patients has more or less stagnated and no significant therapy improvements have been achieved (4).

Massive research in the field of OS is ongoing to assess the prognostic and therapeutic impact of possible biomarkers and altered molecular pathways. For instance, several studies detected frequent genomic alterations of the tumor suppressor genes TP53 and RB1 in OS and correlated these findings with disease outcome (5–7). Other studies identified p-glycoprotein and ezrin that influence the response to chemotherapy and metastatic spread, respectively (8). Recently, attention has been paid to the value of small non-coding microRNAs in the pathogenesis of OS, e.g. the miR-17∼92 cluster (9, 10) and miR-9-5p (11, 12). MicroRNAs represent interesting biomarkers for OS, as they are able to simultaneously regulate hundreds of target genes and several molecular pathways (13).However, the prognostic and therapeutic significance neither for distinct genes including their gene products nor for microRNAs has been determined in controlled clinical studies yet (3). The key prognostic determinants are still clinico-pathological factors and include tumor stage (14), patient age, tumor size and location and the response to neoadjuvant chemotherapy (15). Consequently, all patients are treated with multiagent chemotherapy irrespective of its individual efficacy (16). Moreover, new studies about OS are continuously published and complicate the acquisition of information for specific research purposes and questions.

To support the efforts in OS research and biomarker discovery, we constructed the Osteosarcoma Database. It provides a structured and review-like overview on current OS knowledge with the possibility to rank and sort the literature according to various parameters, including therapeutic and prognostic value of specific genes and microRNAs and the type of samples used. Information of genes and microRNAs in OS was collected by automated literature mining and manual review and annotation of PubMed abstracts. This information was further enriched by determining microRNA–target gene interactions (MTIs) of all collected candidates related to OS.

## Database Construction

**Figure 1.**
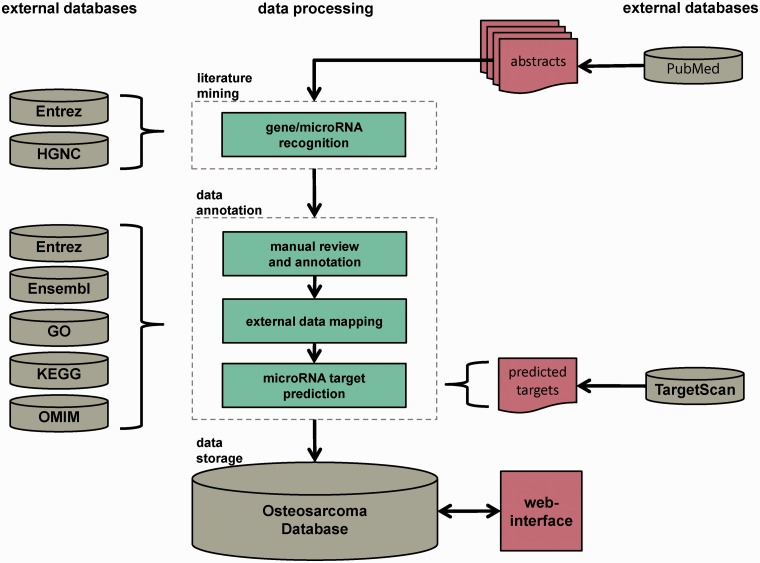
Database construction pipeline. The database construction is performed in three major steps: automated dictionary-based literature mining, data review and annotation by reviewers and external data sources and data storage in a MySQL relational database with Web interface. The whole pipeline is based on PubMed-derived abstracts related to OS research.

The Osteosarcoma Database aims to provide a high-quality collection of genes and microRNAs implicated in the pathogenesis of OS, reviewed by experts of the field. The data collection and processing steps are illustrated in Figure 1. The workflow comprised three major steps: automated dictionary-based gene and microRNA recognition, manual review and annotation and data storage. The pipeline was based on PubMed abstracts that contained the keywords ‘osteosarcoma*’ or ‘osteogenic+sarcoma*’ in their titles and/or abstracts. They were downloaded with the R package XML (17) via NCBI’s E-utilities. Only abstracts written in English and involving human data or specimens were considered. The last download of abstracts was executed on 29 October 2013. In total, 9908 PubMed abstracts were obtained and served as initial corpus for further processing.

### Dictionary-based gene and microRNA recognition

To reduce the time-consuming process of manual review and annotation, a dictionary-based gene and microRNA recognition was performed on the initial corpus of abstracts.

The dictionary of human genes was compiled from the Human Genome Organisation (HUGO) gene nomenclature committee (18) and the National Center for Biotechnology Information (NCBI) Entrez gene database (19). Official symbols, aliases, synonyms, descriptions, names and database accessions of all genes were combined to generate the gene dictionary with the Entrez geneid as unique identifier. The gene dictionary was extended by textual variants of genes (e.g. IL6, IL 6 or IL-6) to be as complete as possible. Ambiguous synonyms and frequent English words according to the stop words function of the R package tm (20) were excluded to avoid inaccurate gene recognitions. In case of microRNAs, regular expressions like ‘mir’, ‘miR’, ‘MIR’, ‘miRNA’ and ‘microRNA’ were used for entity recognition. The miRBase (21) accessions of mature microRNA sequences served as unique identifiers.

Genes included in the dictionary were identified in the initial corpus of abstracts by string matching and the microRNAs by regular expressions using the R package tm (20). Abstracts without any gene or microRNA occurrence were excluded from further processing, e.g. abstracts of epidemiologic studies. The remaining abstracts were manually reviewed and annotated according to their functional role in the OS.

### Manual review and annotation

During the manual review and annotation step, the reviewers verified the specific genes and microRNAs recognized in the abstracts. Additionally, information about experimental settings, the biological context and therapeutic and prognostic impact was marked. The experimental settings comprised the experimental procedure, name of cell lines and kind of samples. Abstracts dealing with human OS cell lines but describing anything but OS biology were excluded.

To provide as much information as possible, we mapped OS-related genes and microRNAs to external databases like NCBI Entrez gene (19), Ensembl (22), Online Mendelian Inheritance in Man (OMIM) (23), Gene Ontology (24), Kyoto Encyclopedia of Genes and Genomes (KEGG) Pathway (25) and miRBase (21). Furthermore, the OS-related literature derived from PubMed (26) was linked to each gene and microRNA entry.

As microRNA regulation has become a major subject of OS research, we determined possible MTIs between OS-related genes and microRNAs. Predicted microRNA targets were computed by running the local perl scripts targetscan_60.pl and targetscan_61_context_scores.pl that were downloaded from the TargetScan Web site (http://www.targetscan.org/) (27). Mature microRNA sequences were gained from miRBase release 20 (21). To obtain high-efficacy targets, we excluded target predictions with a context score > −0.1 (27).

### Data storage

To store and access the collected information on OS-related genes including their gene products and microRNAs, we implemented a database and a user-friendly Web interface. The Osteosarcoma Database is a MySQL relational database. The database scheme is illustrated in Supplementary Figure S1. To easily access OS-related genes and microRNAs, users can search and browse via a Web interface at http://osteosarcoma-db.uni-muenster.de. It is built on PHP and JavaScript. For interactive data visualization, we applied tagcanvas (http://www.goat1000.com/tagcanvas.php) and cytoscapeweb (28). Alternatively, users can download the Osteosarcoma Database sql file to perform their own queries. The download link is provided at http://osteosarcoma-db.uni-muenster.de/download.php.

## Database Description

The Osteosarcoma Database allows retrieving information of candidate genes including their gene products and microRNAs associated with the pathogenesis of OS to support their individual research purposes. Beside gene and microRNA information derived from external databases, manual annotations of OS-related abstracts are provided. Annotations include the number of abstracts focusing on the specific genes with their gene products and microRNAs, the experimental procedures conducted in distinct studies, the potential therapeutic and prognostic value of genes and microRNAs, the specific data types and the biological context investigated. Additionally, regulatory MTIs between collected microRNAs and genes were added. Currently, the database contains 911 genes including their gene products and 81 microRNAs associated with osteosarcoma biology according to 1331 abstracts. Between these microRNAs and genes, we determined 6305 regulatory MTIs due to TargetScan 6 (27).

The database can be searched using the Web interface (http://osteosarcoma-db.uni-muenster.de) with two possible input forms depending on the user’s research focus. For gene search, Entrez geneids and official gene symbols are accepted. MicroRNAs require miRBase accessions or names of mature microRNA sequences. A search for word components is also possible. After submitting the query, suggestions of genes or microRNAs are presented matching the search term. Users can select their requested entry and the results page is displayed.

**Figure 2.**
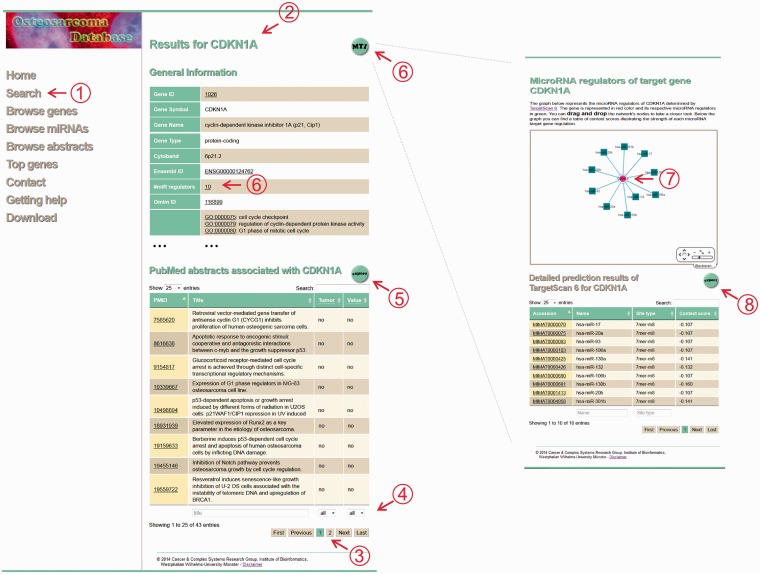
Screenshot of the CDKN1A results page. The database screenshots show the main results page of a gene search and the corresponding MTI network using the example of CDKN1A. (1) The search menu enables the user to search for a gene or microRNA query. (2) Submitting the query delivers the results page for the specific query that shows general information derived from external databases and abstracts associated with the query. (3) The table of abstracts can be browsed using pagination buttons and (4) filtered according to type of samples, potential prognostic and/or therapeutic value or text search within the titles. (5) To receive more manual annotations like experimental settings, biological context and information about the abstracts, an export button is provided. (6 + 7) The MTI network visually illustrates the possible regulatory relationships of the user’s query. A detailed description of the prediction results is given in the table below. (8) Again, users are able to export the table and receive additional information like UTR coordinates and so on.

The main results page lists general information of the requested gene or microRNA. Underscored entries provide links to respective external databases. Below the general gene or microRNA information, a table marks the abstracts describing the gene’s or microRNA’s involvement in the pathogenesis of OS. The abstracts can be filtered according to potential therapeutic and prognostic value and according to tumor samples. Further annotation of experimental settings and biological contexts is provided for download using the export button on top of the table. To note, even if the selection of abstracts was initially based on gene names, we also included experiments involving their gene products such as immunohistochemistry and western blots. However, gene symbols are used as unique identifiers for each gene and/or gene product. Moreover, regulatory MTIs of a specific query are accessible via the MTI button on top of the results page. This button directs the user to predicted microRNA target gene networks. For microRNAs, all target genes are visualized, and for genes, the microRNAs that regulate the respective genes are presented. The network can be explored by zooming in and out or drag and drop nodes. Below the network, details of TargetScan predictions are given. Figure 2 illustrates the main results page and the MTI network using the example of the gene CDKN1A.

Alternatively, the user can browse collected genes, microRNAs and abstracts stored in the database. The last column of all browse tables provides a link to the main results page of the respective gene or microRNA. To visually explore genes including gene products frequently mentioned in OS-related literature, a tagcloud of the top genes was implemented. Just genes mentioned in at least five PubMed abstracts are visualized as top genes. By clicking on gene names, the user is again directed to the main results page for the specific gene.

If we miss specific genes or publications about osteosarcoma, users are welcome to suggest them to us via a contact form, and we are pleased to add them to the database. A graphical guide through the Osteosarcoma Database is available for download on the database Web site at http://osteosarcoma-db.uni-muenster.de/php/tutorial.pdf.

## Discussion and Future Directions

The ongoing research to detect genes or pathways frequently altered in OS and the search for new therapeutic and prognostic procedures is hampered by the genetic complexity of OS. It becomes even more complicated because of the ever increasing literature about studies of OS that make literature research highly time-consuming. Therefore, it is necessary to structure the existing knowledge of genes and microRNAs associated with OS. On that account, we developed the Osteosarcoma Database to supply a review of the current state of OS research and made this information easily accessible to researchers.

### Pathway enrichment analysis on osteosarcoma-related genes

To evaluate the content of the Osteosarcoma Database regarding its functional association to cancer, we performed a KEGG pathway enrichment analysis. All Entrez genes in the human genome were used as a background set. The hypergeometric test was computed to find significantly overrepresented categories (false discovery rate <0.05). The top 20 enriched pathways are listed in Table 1.

**Table 1.** KEGG pathway enrichment analysis

ID	KEGG pathway	Number of genes	Number of genes in pathway	*P*-value	FDR^a^
hsa05200	Pathways in cancer	158	327	5.74 × 10^–48^	1.11 × 10^–45^
hsa05215	Prostate cancer	59	89	8.83 × 10^–28^	8.57 × 10^–26^
hsa05219	Bladder cancer	33	42	9.62 × 10^–20^	6.22 × 10^–18^
hsa05212	Pancreatic cancer	44	70	1.30 × 10^–19^	6.31 × 10^–18^
hsa04510	Focal adhesion	82	200	4.58 × 10^–19^	1.78 × 10^–17^
hsa05222	Small-cell lung cancer	46	85	8.39 × 10^–17^	2.62 × 10^–15^
hsa05220	Chronic myeloid leukemia	42	73	9.45 × 10^–17^	2.62 × 10^–15^
hsa05210	Colorectal cancer	38	62	1.46 × 10^–16^	3.55 × 10^–15^
hsa04110	Cell cycle	58	128	3.74 × 10^–16^	8.07 × 10^–15^
hsa04350	TGF-beta signaling pathway	44	85	3.55 × 10^–15^	6.89 × 10^–14^
hsa05223	Non-small-cell lung cancer	33	54	1.72 × 10^–14^	3.04 × 10^–13^
hsa04115	p53 signaling pathway	38	69	2.01 × 10^–14^	3.25 × 10^–13^
hsa04210	Apoptosis	44	89	3.12 × 10^–14^	4.66 × 10^–13^
hsa05214	Glioma	36	65	7.85 × 10^–14^	1.09 × 10^–12^
hsa05213	Endometrial cancer	31	52	2.86 × 10^–13^	3.70 × 10^–12^
hsa05218	Melanoma	37	71	4.46 × 10^–13^	5.41 × 10^–12^
hsa05142	Chagas’ disease (American trypanosomiasis)	46	104	1.37 × 10^–11^	1.57 × 10^–11^
hsa05221	Acute myeloid leukemia	31	58	1.53 × 10^–11^	1.65 × 10^–10^
hsa04380	Osteoclast differentiation	50	128	4.04 × 10^–11^	4.12 × 10^–10^
hsa04012	ErbB signaling pathway	39	87	4.39 × 10^–11^	4.26 × 10^–10^

The table shows the results of the hypergeometric test of KEGG pathways.

^a^FDR, false discovery rate.

The enrichment results show that the collected OS genes are overrepresented in cancer-related pathways. This indicates that in OS, many well-known oncogenes (e.g. MYC) and tumor suppressor genes (e.g. TP53 and PTEN) are altered. Furthermore, the TGFB signaling pathway is discussed for its contribution to tumor suppression and progression, (29) and the terms apoptosis, cell cycle and focal adhesion represent key signaling pathways in cancer (hallmarks of cancer) (30). Interestingly, we also detected the osteoclast differentiation pathway. In a normal bone, there is a precisely regulated balance between osteoclastic and osteoblastic activity. In OS, this critical balance might be interrupted (31). Taken together, these results indicate OS to require pathways commonly deregulated in cancer as well as to feature OS-specific alterations comprising deregulated osteoclast differentiation.

All properties of OS mentioned earlier are included in the Osteosarcoma Database in terms of OS-related genes, supporting the quality of this collection.

### Prognostic or therapeutic value of genes and microRNAs in osteosarcoma

The ultimate aim of OS research is to understand the molecular mechanism underlying OS biology that would imply the discovery of innovative prognostic and/or predictive biomarkers. The Osteosarcoma Database provides a table that lists the prognostic and/or therapeutic value of genes or microRNAs in corresponding PubMed abstracts. This table can be ranked according to genes or microRNAs with possible impact. Table 2 presents genes and microRNAs that might serve as potential biomarkers in OS. Only genes proposed as candidate markers in at least five studies are listed. As microRNA research is still a young field of research, we list all microRNAs with potential prognostic and predictive impact.

**Table 2.** Most frequent genes and microRNAs with potential therapeutic/prognostic impact

ID	Symbol/Name	Number of abstracts
7157	TP53	26
7422	VEGFA	24
5243	ABCB1	20
2064	ERBB2	14
4193	MDM2	14
5925	RB1	14
7430	EZR	12
249	ALPL	9
1029	CDKN2A	9
632	BGLAP	8
1019	CDK4	8
4609	MYC	7
6678	SPARC	7
595	CCND1	6
4313	MMP2	6
4318	MMP9	6
5743	PTGS2	6
1956	EGFR	5
2353	FOS	5
3939	LDHA	5
4233	MET	5
4288	MKI67	5
MIMAT0000076	hsa-miR-21-5p	2
MIMAT0000092	hsa-miR-92a-3p	1
MIMAT0000232	hsa-miR-199a-3p	1
MIMAT0000267	hsa-miR-210-3p	1
MIMAT0000426	hsa-miR-132-3p	1
MIMAT0000435	hsa-miR-143-3p	1
MIMAT0000447	hsa-miR-134-5p	1
MIMAT0000459	hsa-miR-193a-3p	1
MIMAT0000686^a^	hsa-miR-34c-5p	1
MIMAT0000689	hsa-miR-99b-5p	1
MIMAT0000737	hsa-miR-382-5p	1
MIMAT0001339	hsa-miR-422a	1
MIMAT0004676^a^	hsa-miR-34b-3p	1

The table lists the number of OS-related abstracts of the most frequently mentioned genes and microRNAs associated with any possible prognostic or therapeutic value. The ID column lists Entrez geneids for genes and miRBase accessions for microRNAs.

^a^miR-34 family.

Alkaline phosphatase (ALPL) and lactate dehydrogenase (LDHA) are the only accepted biomarkers with prognostic significance, detectable in the peripheral blood. Concentrations correlate with tumor burden and an adverse outcome (32, 33). Nevertheless, the remaining genes and microRNAs are equally promising candidate markers. For instance, the genes including their gene products EZR and VEGFA are significantly correlated with metastatic spread (8, 34), and the ABCB1 gene coding for the p-glycoprotein seems to be associated with multiple–drug-resistance (8). Additionally, the table shows two members of the microRNA family microRNA-34. These family members are well-characterized tumor suppressors in many cancers and activate TP53 regulated pathways. This microRNA family was extensively tested for its therapeutic use in several tumors and might be the first microRNA family to reach the clinic (35).

Up to now, the prognostic prediction or therapeutic stratification of OS is not based on biomarkers. However, the table suggests many promising candidates that should be further investigated and sometime enter clinical studies.

### Osteosarcoma-related microRNA target gene regulation

Much attention has been focused on microRNAs in the pathogenesis of OS as a new tool for assisting prognosis or therapy. They function through multiple pathways simultaneously, which is in accordance with the perspective on cancer as a disease affecting the whole cellular system. For the collected data, we determined potential MTIs by using TargetScan 6 (27). All microRNAs affecting the largest number of genes (≥100 targets) are shown in Table 3. Again, members of the microRNA family mircoRNA-34 are listed in the table. They regulate the highest number of target genes collected in the Osteosarcoma Database supporting a crucial role in OS as well as in other cancer types. Further, the remaining microRNAs are also known to function as tumor suppressors or oncomirs, e.g. the microRNA families microRNA-29 and -15. Both families have several members involved in various cancer subtypes (36, 37).

**Table 3.** Top OS-related microRNAs

ID	Name	MTI^a^
MIMAT0000255^b^	hsa-miR-34a-5p	139
MIMAT0000686^b^	hsa-miR-34c-5p	138
MIMAT0000271	hsa-miR-214-3p	128
MIMAT0000430	hsa-miR-138-5p	127
MIMAT0000080	hsa-miR-24-3p	126
MIMAT0000068^c^	hsa-miR-15a-5p	122
MIMAT0000417^c^	hsa-miR-15b-5p	121
MIMAT0000100^d^	hsa-miR-29b-3p	119
MIMAT0002820^c^	hsa-miR-497-5p	118
MIMAT0000084	hsa-miR-27a-3p	117
MIMAT0000086^d^	hsa-miR-29a-3p	117
MIMAT0000461^c^	hsa-miR-195-5p	117
MIMAT0000069^c^	hsa-miR-16-5p	116
MIMAT0000763	hsa-miR-338-3p	116
MIMAT0000231	hsa-miR-199a-5p	110
MIMAT0000423	hsa-miR-125b-5p	106
MIMAT0000261	hsa-miR-183-5p	100
MIMAT0000691	hsa-miR-130b-3p	100

The table illustrates the microRNAs regulating most of the genes in the Osteosarcoma Database. All microRNAs regulating ≥100 targets are denoted. The ID column lists miRBase accessions for mature microRNAs.

^a^MTI, microRNA–target gene interaction.

^b^miR-34 family.

^c^miR-15 family.

^d^miR-29 family.

As already mentioned, microRNA research is a young field and not much is known about their function in OS. Thus, we provide detailed and up-to-date networks about possible MTIs to researchers for hypothesis generation and testing of individual models.

### Future directions

Currently, the Osteosarcoma Database focuses on genes including their gene products and microRNAs associated with OS development and progression. However, the OS is a complex tumor with a huge amount of genomic instability that influences the expression and function of several genes and microRNAs. Hence, genomic alterations need to be added in future versions. We plan to include already known genomic positions marking regions of copy number variations, allelic imbalances and translocations, as it has been shown that structural chromosomal alterations could be used to predict prognosis at diagnosis (2). Moreover, observations of genome-wide changes from next-generation sequencing studies might further obtain new insights into OS biology and must be added as soon as they are available.

We plan to update the database biannually to provide state-of-the-art knowledge and keep track of improvements in the field. We hope that the Osteosarcoma Database will serve as a platform for information and hypothesis generation for the research community that helps to uncover the complexity of OS.

## Supplementary data

Supplementary Data are available at *Database* Online.
